# The Potential Effects of Therapist Ageism on Older Adults’ Therapy Outcomes: A Scoping Review

**DOI:** 10.1093/geroni/igaf122.2213

**Published:** 2025-12-31

**Authors:** Lea Beresford, Richard Zweig

**Affiliations:** Ferkauf Graduate School of Psychology, Bronx, New York, United States; Ferkauf Graduate School of Psychology, Bronx, New York, United States

## Abstract

Ageism has profound effects on the mental health of older adults. Further, psychotherapists are not immune to holding ageist beliefs and ageism in therapists may affect older adults’ therapy outcomes. Data selection included searches (using key words e.g. “ageism,” “aging,” “age stereotype,” “older adult,” “access,” “therapeutic alliance,” “prognosis,” and “therapy outcome”) across Google Scholar and PsycInfo from 2000 on and yielded 15 studies about older adults’ experience of ageism, therapists’ attitudes toward treating older adults, and therapists’ expectations about therapy with older adults. Analogue studies of psychotherapists indicated that they were less willing to treat older adults. Analogue and survey studies of therapist biases and rapport suggested that psychotherapists who held stereotypical attitudes were less willing to work with the subjects of their beliefs and had worse expectations for the therapeutic alliance, while the patients reported poorer working relationships with these therapists. Analogue studies of therapists revealed that they treated older adults differently by offering dissimilar, potentially less effective therapies and expected poorer prognoses and outcomes. A major limitation is that analogue studies may not accurately predict attitudes and behaviors in the real world. In conclusion, ageism in therapists may negatively affect older adults’ access to therapy, therapists’ alliances with older adult patients, the type and quality of therapy therapists offer older adults, and therapists’ expectations of older adults’ prognoses and outcomes.

